# Supplementary material for: Angiotensin-converting enzyme polymorphisms AND Alzheimer’s disease susceptibility: An updated meta-analysis
Source: PLoS One. 2021 Nov 24;16(11):e0260498. doi: 10.1371/journal.pone.0260498 (PMC8612529; doi:10.1371/journal.pone.0260498)
Supplement: Attachment — Submitted filename: Response to reviewers.docx [file pone.0260498.s002.docx]

Replies to Academic Editor

We are very grateful for your careful work earnestly. We considered every comment and suggestion of you and made cautious revision accordingly as follows:

**Comment 1**: Please ensure that your manuscript meets PLOS ONE's style requirements, including those for file naming.

**Response**: We have revised our manuscript according to the PLOS ONE style templates to make it meet the style requirements, including those for file naming.

**Comment 2**: Please include the tables to within the manuscript.

**Response**: The tables have been included within the manuscript.

**Comment 3**: Please change ‘Caucasian’to ‘white’ or ‘of European descent’.

**Response**: According to your suggestion, ‘of European descent’ or ‘Europeans’ has been used to substitute ‘Caucasian’ in the revised manuscript.

**Comment 4**: Please ensure that you provide the correct grant numbers for the awards you received for your study in the ‘Funding Information’ section.

**Response**: Thank you for your careful checks. We have corrected the grant number in ‘Funding Information’ section according to your positive comment.

**Comment 5**: Please amend your list of authors on the manuscript to ensure that each author is linked to an affiliation.

**Response**: We have amended our list of authors to ensure that each author is linked to an affiliation in the revised version.

**Comment 6**: Please include captions for your Supporting Information files at the end of your manuscript, and update any in-text citations to match accordingly.

**Response**: We have added captions for Supporting Information files at the end of our manuscript and updated in-text citations to match accordingly.

Replies to Reviewer 1

We would like to express our sincere thanks to you for your valuable suggestions and comments, which enable us to greatly improve the quality of our manuscript. The revised parts that correspond to the comments are listed as follows:

**Comment 1:** Some viewpoints need more supporting materials or references, such as “the ε4 allele accounts for an estimated 45% to 60% of the genetic susceptibility to AD”

**Response**: We totally agree with your suggestion. The statement of “the *ε4* allele accounts for an estimated 45% to 60% of the genetic susceptibility to AD” has been corrected. We rewrote this part as “Presence of APOE *ε4* allele increases risk of AD with a dose-dependent manner, and might lead to an earlier age of disease onset. The frequency of APOE *ε4* in AD patients varied among different ethnics groups, ranging from around 40% to 60%, compared to 20%~25% in controls.” We think this modification help us express our viewpoint more clearly and accurately. In addition, we also added two references to support this viewpoint. (seen in the 2^nd^ paragraph of Introduction)

1. Alex Ward, Sheila Crean, Catherine J Mercaldi, Jenna M Collins, Dylan Boyd, Michael N Cook, et al. Prevalence of apolipoprotein E4 genotype and homozygotes (APOE 4/4) among patients diagnosed with Alzheimer's disease: a systematic review and meta-analysis. Neuroepidemiology. 2012;38(1):1-17. DOI: [10.1159/000334607](https://doi.org/10.1159/000334607). PMID: 22179327. (Reference 4 in the manuscript)

2. Verghese PB, Castellano JM, Holtzman DM. [Apolipoprotein E in Alzheimer's disease and other neurological disorders.](https://pubmed.ncbi.nlm.nih.gov/21349439/) Lancet Neurol. 2011;10(3):241-52. DOI: [10.1016/S1474-4422(10)70325-2](https://doi.org/10.1016/s1474-4422(10)70325-2). PMID: 21349439. (Reference 5 in the manuscript)

**Comment 2**: “Angiotensin converting enzyme (ACE) is an important component of the renin-angiotensin system (RAS), which mainly acts on promoting the formation of Angiotensin II (Ang II) from Angiotensin I (Ang I). Recently, many evidences supported that ACE participated in the pathogenesis of AD. As a membrane-bound zinc metalloprotease, ACE played an important role in Aβ degradation.” should be revised as “Recently, many evidences supported that ACE participated in the pathogenesis of AD. As a membrane-bound zinc metalloprotease, ACE played an important role in Aβ degradation. Angiotensin converting enzyme (ACE) is an important component of the renin-angiotensin system (RAS), which mainly acts on promoting the formation of Angiotensin II (Ang II) from Angiotensin I (Ang I).”.

**Response**: We appreciate your detailed comment. We have revised this part according to your kind suggestion. Now It reads more fluent and logistic. (seen in the 3^rd^ paragraph of Introduction)

**Comment 3**: Some sentences were obscure, like “We also stratified the study cohorts, when possible, according to the age of onset, ethnic background and APOE *ε4* status.” The relationship between ACE and APOE *ε4* should be illustrated more.

**Response**: We agree with your assessment. We have incorporated your comment in the last paragraph of the introduction part. We added two references to support the possible relationship between ACE and APOE *ε4*, as well as to clarify why we stratified the study cohorts according to APOE *ε4* status: “Since some recent evidence suggested that the presence of APOE *ε4* influence the behavioural effects of ACE I/D polymorphism in AD, and the protective effects of ACE inhibitors or angiotensin receptor blockers on cognitive decline correlated with APOE *ε4* carrier status, we also performed subgroup analyses according to APOE *ε4* carrier status if sufficient data could be obtained”. (seen in the last paragraph of Introduction)

1. Oliveira FF, de Almeida SS, Smith MC, Bertolucci PHF. Behavioural effects of the ACE insertion/deletion polymorphism in Alzheimer's disease depend upon stratification according to APOE-*ε4* carrier status. Cogn Neuropsychiatry. 2021;26(4):293-305. DOI: [10.1080/13546805.2021.1931085](https://doi.org/10.1080/13546805.2021.1931085). PMID: 34034613 (Reference 12 in the manuscript)

2. Ouk M, Wu CY, Rabin JS, Jackson A, Edwards JD, Ramirez J, The use of angiotensin-converting enzyme inhibitors vs. angiotensin receptor blockers and cognitive decline in Alzheimer's disease: the importance of blood-brain barrier penetration and APOE *ε4* carrier status. Alzheimers Res Ther. 2021 Feb 11;13(1):43. DOI: [10.1186/s13195-021-00778-8](https://doi.org/10.1186/s13195-021-00778-8). PMID: 33573702 (Reference 13 in the manuscript)

**Comment 4**: Are there any RCTs (the most convincing type in meta-analysis) involved in this research? If yes, pls add one or two sentences in “study election”. If not, pls also explain the reason.
**Response**: You have raised an important concern, and we believe that RCTs are the most convincing type in meta-analysis. However, randomized controlled design might be more suitable for clinical trials rather than gene polymorphism studies of patients and controls. We could not identify any RCTs in electronic database search in the present investigation. We have added illustration to this point as follows in Study selection: “Since all the included studies were gene polymorphism investigations in patients and controls, which were not suitable for randomized controlled design, no randomized controlled trial (RCT) could be identified and involved in our research.” (seen in the 1^st^ paragraph of Study selection in Materials and methods)

**Comment 5**: In the conclusion, the sentence “While rs4343, rs4291 and rs4309 polymorphisms are unlikely to be major factors in AD development” sounds kind of absolute. It could be better when add “in our/this research” in the end.

**Response**: We agree with your advice. We have added the words “in our research” in the end of this sentence in the revised manuscript. (seen in Conclusions)

Sincerely yours,

Xiao-Yu Xin

Replies to Reviewer 2

Thanks a lot for your generous and detailed comments. Your suggestions provide an important direction for us to revise our paper. We itemized our point-by-point responses to your comments as follows:

**Comment 1**: Please identify what is I and D in the Abstract.

**Response**: Thank you for your reminding, we have identified what is I and D in Abstract in the revised version: “the insertion (*I)* allele conferred increased risk to AD compared to the deletion (*D)* allele”. (seen in Abstract)

**Comment 2**: In the introduction, the authors described “However, the *ε4* allele accounts for an estimated 45% to 60% of the genetic susceptibility to AD, which is neither necessary nor sufficient to cause the disease”. But there are still some references supporting APOE *ε4* which increases risk for Alzheimer's disease and is also associated with an earlier age of disease onset. Please clarify.
**Response**: Thank you for pointing out the potential for misunderstanding. We have adjusted the text to be clearer. According to your nice suggestion, we revised this part as follows: “Presence of APOE *ε4* allele increases risk of AD with a dose-dependent manner, and might lead to an earlier age of disease onset. The frequency of APOE *ε4* in AD patients varied among different ethnic groups, ranging from around 40% to 60%, compared to 20%~25% in controls. Therefore, the presence of *ε4* is neither necessary nor sufficient to cause the disease, indicating the participant of other heritable risk factors underlying the development of AD”. We also added two references to support the above statements. (seen in the 2^nd^ paragraph of Introduction)

1. Alex Ward, Sheila Crean, Catherine J Mercaldi, Jenna M Collins, Dylan Boyd, Michael N Cook, et al. Prevalence of apolipoprotein E4 genotype and homozygotes (APOE 4/4) among patients diagnosed with Alzheimer's disease: a systematic review and meta-analysis. Neuroepidemiology. 2012;38(1):1-17. DOI: [10.1159/000334607](https://doi.org/10.1159/000334607). PMID: 22179327. (Reference 4 in the manuscript)

2. Verghese PB, Castellano JM, Holtzman DM. [Apolipoprotein E in Alzheimer's disease and other neurological disorders.](https://pubmed.ncbi.nlm.nih.gov/21349439/) Lancet Neurol. 2011;10(3):241-52. DOI: [10.1016/S1474-4422(10)70325-2](https://doi.org/10.1016/s1474-4422(10)70325-2). PMID: 21349439. (Reference 5 in the manuscript)

**Comment 3**: In literature search, the authors indicated that no language restriction. However, normally a better selection criterion is the literature written in English.
**Response**: You have raised an interesting concern. We believe that high quality investigations usually published in English. However, the strength of meta-analysis depends on combining all available published data to increase statistical power, including those not written in English. Therefore, beside PubMed, Embase and Alzgene, we also searched CNKI (China National Knowledge Infrastructure) for associated studies, which mainly contains literature written in Chinese. Moreover, some high- quality meta-analyses used CNKI for literature search too, such as those published in Lancet series [1,2]. In addition, we used Newcastle-Ottawa Scale (NOS) to assess the methodological quality of each involved study, which has been widely used in meta-analysis (Table 1) [3]. In our analyses for rs1799752, 7 studies were published in Chinese, and others in English. According to your nice suggestion, after broad analysis, we performed analyses particularly in studies written in English. Similar results were obtained after excluding literature in Chinese. We supplied the above results in our revised manuscript: “When analyses were performed particularly in investigations published in English, no reliable associations were identified either.” (seen in the 1^st^ paragraph of rs1799752 I/D and AD risk in Results). For the other several polymorphisms, all the included investigations were written in English. (seen in the 1^st^ paragraph of rs1800764 T/C and AD risk in Results, the 1^st^ paragraph of Other polymorphisms and AD risk in Results)

1. Slee A, Nazareth I, Bondaronek P, Liu Y, Cheng Z, Freemantle N. Pharmacological treatments for generalised anxiety disorder: a systematic review and network meta-analysis. Lancet. 2019;393(10173):768-777. DOI: [10.1016/S0140-6736(18)31793-8](https://doi.org/10.1016/s0140-6736(18)31793-8). PMID: 30712879

2. Funk AL, Lu Y, Yoshida K, Zhao T, Boucheron P, van Holten J, et al. Efficacy and safety of antiviral prophylaxis during pregnancy to prevent mother-to-child transmission of hepatitis B virus: a systematic review and meta-analysis. Lancet Infect Dis. 2021;21(1):70-84. DOI: [10.1016/S1473-3099(20)30586-7](https://doi.org/10.1016/s1473-3099(20)30586-7). PMID: 32805200

3. Stang A. [Critical evaluation of the Newcastle-Ottawa scale for the assessment of the quality of nonrandomized studies in meta-analyses.](https://pubmed.ncbi.nlm.nih.gov/20652370/) Eur J Epidemiol. 2010;25(9):603-5. DOI:[10.1007/s10654-010-9491-z](https://doi.org/10.1007/s10654-010-9491-z). PMID:20652370 (Reference 14 in the manuscript)

**Comment 4**: In study selection, the authors should try to select the cohort with higher numbers of subjects. Because they mentioned in the introduction that “some discrepancies may be related to the small sample size of individual studies”.
**Response**: Thank you for pointing out the ambiguous statement in our article. We meant that small sample size, in combination with modest effect, might contribute to low statistical power, which was an important reason responsible for the diversity of previous results. None of individual studies could show more sufficient statistical power than the results of meta-analysis. Because the strength of meta-analysis is the accumulation of all the available published data to increase statistical power, largely addressing the issue of sample size. We have adjusted the text to be clearer as follows: “While meta-analysis is a well-established means to quantitatively synthesize all association data across studies to reduce heterogeneity and identify minor genetic effects, which largely addressing the issue of sample size.” (seen in the 4^th^ paragraph of Introduction). In addition, according to your comment, we compared pooled ORs of large sample size subgroup (more than 300 cases and controls in total) and small sample size subgroup (less than 300 cases and controls in total) when possible, but no significant difference was identified. We also added this part to the revised manuscript. (seen in the last paragraph of Statistical analysis in Materials and methods, the 2^nd^ paragraph of rs1799752 I/D and AD risk in Results, the 1^st^ paragraph of rs1800764 T/C and AD risk in Results, the 1^st^ paragraph of Other polymorphisms and AD risk in Results)

**Comment 5**: The authors should try to link the correlation between rs1799752 polymorphism or others and their potential effects on the function of ACE.

**Response**: Thank you for your kind reminding. We have added the potential effects of rs1700752 polymorphism on ACE function in the 4^th^ paragraph of introduction as follows: “The I/D genotype is regarded as a determinant of ACE expression levels in plasma, cells and tissues. Approximately 50% variability in plasma levels of ACE depends on the rs1799752 polymorphism”. We also added 2 references to support the possible correlation. Unfortunately, we found few studies reporting the functions of other polymorphisms. We only know that “rs1800764 and rs4291 located in the regulatory region of ACE gene, while rs4343 in the exotic region.” Though function of the above polymorphisms is outside our investigation, we believe that you have raised a significant direction in future consideration. (seen in the 4^th^ paragraph of Introduction)

1. Cafiero C, Rosapepe F, Palmirotta R, Re A, Ottaiano MP, Benincasa G, et al. Angiotensin System Polymorphisms' in SARS-CoV-2 Positive Patients: Assessment Between Symptomatic and Asymptomatic Patients: A Pilot Study. Pharmgenomics Pers Med. 2021;14:621-629. DOI: [10.2147/ PGPM.S303666](https://doi.org/10.2147/pgpm.s303666). PMID: 34079337. (Reference 9 in the manuscript)

2. Ghafouri-Fard S, Noroozi R, Omrani MD, Branicki W, Pośpiech E, Sayad A, et al. Angiotensin converting enzyme: A review on expression profile and its association with human disorders with special focus on SARS-CoV-2 infection. Vascul Pharmacol. 2020;130:106680. DOI: [10.1016/j.vph.2020. 106680](https://doi.org/10.1016/j.vph.2020.106680). PMID: 32423553. (Reference 10 in the manuscript)

**Comment 6**: It is suggested to discuss why North Europeans compared to East Asians carrying rs1799752 polymorphism are more susceptible to AD occurrence.

**Response**: We think that ethnic genetic background difference is the main reason responsible for the difference of pooled ORs between North Europeans and East Asians. For example, the prevalence of *I* allele in Asian controls is higher than in European controls not only in our study but also in a recent report [1]. But the underlying reason hasn’t been identified yet, which still need further investigation to clarify. Thanks to your constructive advice, we have added this part in the 5^th^ paragraph of Discussion as follows: “We attributed the difference of results among Europeans and Asians mainly to the difference of genetic background. However, no comparable investigations have been carried out with regard to mechanisms underlying rs1799752 polymorphism and AD development between different ethnics, which still need further research to clarify.”(seen in the 5^th^ paragraph of Discussion)

1. Hatami N, Ahi S, Sadeghinikoo A, Foroughian M, Javdani F, Kalani N, et al. Worldwide ACE (I/D) polymorphism may affect COVID-19 recovery rate: an ecological meta-regression. Endocrine. 2020;68(3):479-484. DOI: 10.1007/s12020-020-02381-7. PMID: 32542429

Best wishes,

Xiao-Yu Xin
